# DNA Probes for Cas12a-Based Assay with Fluorescence Anisotropy Enhanced Due to Anchors and Salts

**DOI:** 10.3390/bios13121034

**Published:** 2023-12-16

**Authors:** Irina V. Safenkova, Alexey V. Samokhvalov, Kseniya V. Serebrennikova, Sergei A. Eremin, Anatoly V. Zherdev, Boris B. Dzantiev

**Affiliations:** 1 A.N. Bach Institute of Biochemistry, Research Centre of Biotechnology of the Russian Academy of Sciences, Moscow 119071, Russia; safenkova@inbi.ras.ru (I.V.S.); a.samohvalov@fbras.ru (A.V.S.); ksenijasereb@mail.ru (K.V.S.); eremin-fbras@yandex.ru (S.A.E.); zherdev@inbi.ras.ru (A.V.Z.); 2 Faculty of Chemistry, M.V. Lomonosov Moscow State University, Moscow 119991, Russia

**Keywords:** CRISPR/Cas12, trans-cleavage, ssDNA probe, DNA trans-target, hairpin probe, G-quadruplex, fluorescence polarization

## Abstract

CRISPR/Cas12a is a potent biosensing tool known for its high specificity in DNA analysis. Cas12a recognizes the target DNA and acquires nuclease activity toward single-stranded DNA (ssDNA) probes. We present a straightforward and versatile approach to transforming common Cas12a-cleavable DNA probes into enhancing tools for fluorescence anisotropy (FA) measurements. Our study involved investigating 13 ssDNA probes with linear and hairpin structures, each featuring fluorescein at one end and a rotation-slowing tool (anchor) at the other. All anchors induced FA changes compared to fluorescein, ranging from 24 to 110 mr. Significant FA increases (up to 180 mr) were obtained by adding divalent metal salts (Mg^2+^, Ca^2+^, Ba^2+^), which influenced the rigidity and compactness of the DNA probes. The specific Cas12a-based recognition of double-stranded DNA (dsDNA) fragments of the bacterial phytopathogen *Erwinia amylovora* allowed us to determine the optimal set (probe structure, anchor, concentration of divalent ion) for FA-based detection. The best sensitivity was obtained using a hairpin structure with dC10 in the loop and streptavidin located near the fluorescein at the stem in the presence of 100 mM Mg^2+^. The detection limit of the dsDNA target was equal to 0.8 pM, which was eight times more sensitive compared to the common fluorescence-based method. The enhancing set ensured detection of single cells of *E. amylovora* per reaction in an analysis based on CRISPR/Cas12a with recombinase polymerase amplification. Our approach is universal and easy to implement. Combining FA with Cas12a offers enhanced sensitivity and signal reliability and could be applied to different DNA and RNA analytes.

## 1. Introduction

Clustered regularly interspaced short palindromic repeats/CRISPR-associated protein (CRISPR/Cas12a) technology is gaining increasing attention as a tool for specific DNA recognition in diagnostic applications [1,2,3]. This technology is built upon the ability of the Cas12a endonuclease, in a complex with guide RNA (gRNA), to initiate two consecutive processes: (1) recognition of the nucleotide sequence (target dsDNA/ssDNA/RNA) complementary to the gRNA and (2) activation of Cas12a’s trans-nuclease capability (trans-cleavage) against any single-stranded DNA (ssDNA) with a length of at least five nucleotides [4,5,6]. The larger the quantity of target nucleic acids is in the sample, the more Cas12a will be activated, resulting in the cleavage of more ssDNA.

This dependence is used in biosensors by introducing labeled ssDNA probes into the sample [7,8]. The most common probes include a fluorescent label and a quencher molecule at opposite ends of the ssDNA. After the trans-cleavage of the ssDNA probe, a detectable fluorescent signal will emerge in the solution [9]. Biosensors, such as DETECTR [5], HOLMES [10], and other Cas12a-based biosensors [7], operate on this principle. The second type involves probes detected with lateral flow test strips (LFTSs). In this case, the ssDNA probe is labeled with tags that are specifically and affinity-recognized on the test strip [11]. There are several variants of probes for LFTS detection differing in functional tags. The most frequent ones are fluorescein and biotin located at opposite ends of the cleaved ssDNA [9]. Other proposed tags are IgG released after trans-cleavage [12] and human chorionic gonadotropin [13]. ssDNA probes of the third group have a detectable label at one end, whereas their other end is attached to a carrier surface: magnetic nanoparticles, polystyrene microplates, etc. Trans-cleavage destroys the ssDNA probe and releases the label from the surface. In this case, the label can be a fluorescent molecule, an enzyme, a nanozyme, or another detectable molecule [14,15,16,17,18].

An intriguing approach is the use of fluorescence anisotropy (FA) for the detection of the probe’s trans-cleavage. FA detection is based on the excitation of the fluorophore attached to the target using plane-polarized light. FA measurements reflect changes in polarization of the light emitted by the fluorophore. When a fluorophore-containing structure changes (increases in size due to interaction with a receptor or, conversely, decreases due to degradation), the ratio of light emitted by the fluorophore along the parallel and perpendicular axes of polarization also changes. Competitive advantages of FA-based assays are determined by the choice of the registered optical parameter and their implementation in one step (mix-and-detect mode). FA is a more efficient and accurate detecting parameter than the commonly applied fluorescence because FA is less dependent on the influence of the sample compounds [19]. Registration of FA as the ratio of two fluorescence intensities allows largely compensating the influence of colored samples on the assay results. In addition, the choice of the fluorophore and tested matrix (for example, the product of the first analytical reaction instead of a biosample) also reduces the absorption of emitted light [20]. The detection of ligand–receptor interactions in a real-time regime, i.e., without enhancing processes, limits the sensitivity of FA-based assays, but this disadvantage is overcome to a certain extent by including the receptor in additional complexes to increase the signal amplitude. FA measurements successfully demonstrate their advantages in assays combining amplification and detection stages. Due to these aforementioned reasons considered, this method is actively used in immunoassays [20,21], and successful examples of its application exist in PCR [22], recombinase polymerase amplification (RPA), and loop-mediated isothermal amplification (LAMP) [23,24]. Note that FA-based assays need instrumental optical measurements and therefore occupy a special niche that distinguishes them from qualitative («yes-no») tests for POC diagnostics with visual assessment of their results, such as immunochromatographic lateral flow tests. In recent years, approaches have been actively developed to simplify and reduce the cost of this registration: portable FA detectors, units for recording FA using serial communication devices [25,26,27,28].

To implement the FA approach, the FA of the uncleaved probe should differ from the FA of the cleaved probe. Trans-cleavage should significantly alter the FA of the fluorophore-containing probe. After the trans-cleavage of the ssDNA probe is completed, the remaining compound with the attached fluorophore is small, rotates rapidly, and demonstrates low FA. Therefore, the initial ssDNA probe must be large, rotate slowly, and exhibit high FA. Several parameters influence the FA of the ssDNA probe, including mass, hydrodynamic dimensions, viscosity, and structural rigidity [19]. To slow down the rotation of the ssDNA probe, an element called an anchor can be introduced. The anchor typically serves to increase mass and hydrodynamic dimensions, enhancing the FA signal. The strategy of using anchors to amplify the FA signal is successfully used in ligand–receptor systems when the receptor–ligand complex labeled with a fluorescent tag does not yield a sufficiently strong signal; an anchor is introduced to enhance the receptor [20]. For CRISPR/Cas12-based biosensors, there is a single system with an anchor, where DNA nanosheets are added to the ssDNA probe with a fluorescent label after the reaction with Cas12a, serving as an anchor for FA [29]. Thus, the anchor concept works using DNA nanosheets, but for application in CRISPR/Cas12a systems, a simpler and universal ssDNA probe with an anchor is in demand to achieve high FA before cleavage and low FA after cleavage. The goal of this study was to find simple and convenient ssDNA probes with anchors for CRISPR/Cas12a biosensors with FA detection.

In this work, we evaluated protein anchors using the example of streptavidin and nucleic acid anchors, differently structured. All these anchors are highly accessible to users and can be easily integrated into CRISPR/Cas12a biosensors. Additionally, based on the anchor concept and the understanding of ssDNA conformation change in the presence of divalent metal ions [30,31], we proposed a simple method to enhance FA by using a high content of divalent metal ions.

## 2. Materials and Methods

### 2.1. Materials

The ssDNA probes (see sequences in Table 1), gRNA, and primers for PCR and RPA (see sequences in Table S1) were custom-synthesized and purified by Syntol (Moscow, Russia). EnGene LbCas12a and the Monarch DNA gel extraction kit were purchased from NEB (Ipswich, MA, USA). Tersus polymerase and dNTP were obtained from Evrogen (Moscow, Russia). Recombinant streptavidin (STR) was obtained from IMTEK (Moscow, Russia). Tris(hydroxymethyl)aminomethane, zinc sulfate heptahydrate, and barium chloride anhydrous were obtained from Sigma-Aldrich (St. Louis, MO, USA); magnesium acetate tetrahydrate from Honeywell (Charlotte, NC, USA); and calcium chloride dihydrate from Scharlau (Barcelona, Spain). All the chemicals were of analytical or chemical reagent grade. All DNA stock solutions were prepared by dissolving lyophilized DNA in nuclease-free water (Evrogen) to a concentration of 100 μM. A NanoDrop2000 micro-volume spectrophotometer (Thermo Fischer Scientific, Waltham, MA, USA) was used for verification of concentrations of oligonucleotides and proteins.

### 2.2. Estimation of Streptavidin Anchor Impact on Fluorescence Anisotropy of ssDNA Probes in the Absence of Metal Salts

Biotinylated ssDNA probes (200 nM of L-T10, 200 nM of L-C10) (see Table 1, lines 4–5) were mixed with 400 nM of STR (ratio 1 (ssDNA) to 2 (STR)) or 66.6 nM (ratio 3 (ssDNA) to 1 (STR)) in 20 mM Tris-HCl, pH 9.0, and incubated for 30 min at room temperature. Next, 2-fold dilutions starting from 100 nM of a probe without an anchor and with an anchor were prepared in non-binding 96-well black microtiter plates (Thermo Scientific NUNC, Roskilde, Denmark), and the final volume of each well was 100 µL.

For L-T10 and L-T10–STR (1:2 ratio), a complementary oligonucleotide (A10; see Table 1, line 13) was added in equimolar concentration relative to L-T10 in 20 mM Tris-HCl, pH 9.0, containing 10 mM Mg(CH_3_COO)_2_ and incubated for 30 min at room temperature.

The microtiter plates were incubated for 5 min and stirred at 300 rpm for 30 s using a CLARIOstar multimode plate reader (BMGLabtech, Ortenberg, Germany). FA and fluorescent intensity were measured using “fluorescence polarization end-point” mode with excitation filter CLA 482 ± 16 nm, dichroic filter LP 504 nm, and emission filter CLA 520 ± 10 nm. The focal height was set to 5.7 mm. The data were analyzed using CLARIOstar MARS 4.0 R2 software and processed using Origin 2019b software (Origin Lab, Northampton, MA, USA).

### 2.3. Estimation of Divalent Cation Salts’ Impact on Fluorescence Anisotropy of ssDNA Probes without and with Anchors

Conjugates of biotinylated ssDNA probes (linear: L-4, L-5, L-C6, L-T10, L-C10, L-A10; hairpin: H-T5, H-T10, H-C10, H-T30; see Table 1, lines 1–11) with STR were prepared beforehand by mixing 100 nM of ssDNA with 200 nM of STR in 50 mM Tris-HCl, pH 9.

Stock solutions of divalent metal salts were diluted to the following concentrations: 2 M for magnesium and calcium and 1 M for barium in 50 mM Tris-HCl, pH 9. Two stock solutions (1 and 0.4 M) were prepared for zinc. Next, a series of 3- and 5-fold dilutions were prepared for magnesium, calcium, barium, and zinc. Subsequently, 50 µL of 50 nM ssDNA probes or their conjugates with STR were added to the wells of non-binding 96-well black microtiter plates containing 50 µL of divalent metal solution.

FA and fluorescent intensity were measured using a CLARIOstar multimode plate reader (BMGLabtech, Germany), as described in Section 2.2.

### 2.4. Circular Dichroism (CD) Spectra Measurements

All measurements were performed using the Chirascan CD spectrometer (Applied Photophysics, Leatherhead, UK) (bandwidth of 1.0 nm, integration time of 2.5 ns). The CD and absorbance of the G-quadruplex probe were obtained in three different buffers: (1) 50 mM Tris-HCl, pH 9; (2) 10 mM Tris-HCl, 50 mM NaCl, 10 mM MgAcetate_2_, pH 7.9; and (3) 50 mM Tris-HCl, 100 mM MgAcetate_2_, pH 9. Accordingly, the G-quadruplex probe was diluted in the buffer to a final concentration of 1 μM. For each buffer, spectra were obtained in the range from 215 to 320 nm. CD was measured as the differential absorbance (ΔA) (mdeg) of left- and right-circularly-polarized light and then recalculated into molar ellipticity (θ) (deg M^−1^ m^−1^) (θ = ΔA × 3298/(L*C), where L is the path length of the cell (cm) and C is the concentration (mol/L)). The data were analyzed using the Pro-Data Chirascan package 4.2 (Applied Photophysics, UK).

### 2.5. Synthesis of dsDNA Cis-Target for Cas12a

PCR to produce the cis-target DNA fragment of the target AMY1267 gene of *Erwinia amylovora* (strain CFBP 1430) was performed according to [32]. Briefly, PCR was carried out using 500 nM of forward and reverse primers, 200 µM dNTP, Tersus polymerase, 5 × 10^8^ CFU/mL of *E. amylovora* CFBP 1430 (from the collection of the All-Russian Plant Quarantine Center, Bykovo, Russia), and the BioRad T100 Thermal Cycler (Hercules, CA, USA) (38 cycles including 30 s of denaturation at 95 °C, 30 s of annealing at 55 °C, and 60 s of elongation at 72 °C). The obtained cis-target DNA was purified using gel electrophoresis (2% agarose in 20 mM Tris-acetate buffer with 0.2 mM EDTA, 8.3 pH) combined with the DNA gel extraction kit (Evrogen). The dsDNA concentration was measured three times using NanoDrop ND-2000.

### 2.6. Comparison of Probes in the Cas12a-Based Assay Using the dsDNA Cis-Target

A Cas12a-based assay was performed using gRNA (60 nM) and Cas12a (60 nM), which were mixed in NEB2.1 buffer and incubated at 25 °C for 10 min. The ssDNA probe (Table 1) (200 nM) and dsDNA cis-target (10 µL) were added to the gRNA–Cas12a mixture. All concentrations of dsDNA were prepared in NEB 2.1 buffer and varied in the concentration range from 0.2 pM to 10 nM. The total volume was 50 µL. The mixtures were incubated for 30 min at 37 °C. Next, the mixtures were placed in a black microplate with 50 µL of salt buffer (100 mM Tris-HCl containing 100 mM CaCl_2_ or 200 mM Mg(CH_3_COO)_2_). After that, the FA of ssDNA probes (Table 1, lines 1–13) and fluorescence for FAM-T15-BHQ1 (Table 1, lines 15) were measured using the CLARIOstar multimode plate reader (BMGLabtech, Germany), as described in Section 2.2.

The data were approximated with the sigmoidal function y = A2 + ((A1 − A2)/(1 + (x/x0)^p^) using Origin 2019b. The limit of detection (LOD) was calculated as the concentration equal to the triple standard deviation above the blank mean. The limit of quantitation (LOQ) was calculated as the concentration equal to five standard deviations above the blank mean.

### 2.7. Estimation of Trans-Cleavage of the Probes Using Polyacrylamide Gel Electrophoresis

Electrophoretic separation of mixtures after the Cas12a-based assay with the dsDNA target (10 nM) and without it was conducted using a mini-Protean system (Bio-Rad Laboratories, Hercules, CA, USA) with a gel size of 8.3 per 7.3 cm and a PowerPac Universal current source (Bio-Rad Laboratories, Hercules, CA, USA). Electrophoresis was carried out in 15% gel with a ratio of acrylamide to methylenebisacrylamide of 29:1 under denaturing conditions (40 mM Tris-acetate buffer, pH 8.3, with 1 mM EDTA and 7 M urea) with 0.75 mm thickness. The gel was polymerized with the addition of 50 μL of freshly prepared 10% (*v*/*v*) ammonium persulfate and 7.5 μL of N,N,N’,N’-tetramethylethylenediamine per 10 mL of gel mix. Samples (ssDNA probes and mixtures after the Cas12a-based assay) were diluted to a concentration of 50 nM of the probe with an equal volume of 40% glycerol/0.01% bromophenol blue/0.01% xylen cyanol in 40 mM Tris-acetate buffer with 0.4 mM EDTA, pH 8.3. Next, samples were loaded directly onto the gel and allowed to run for 1 h at a constant power of 2 W. The fluorescence of fluorescein at the end of the ssDNA probe was recorded using a ChemiDoc XRS + recording camera (BIORAD, USA). The obtained images were processed using Image Lab 6.0.1 software (Bio-Rad Laboratories).

### 2.8. Cas12a-Based Assay of E. amylovora without Preamplification

A Cas12a-based assay was performed, as described before (see Section 2.6), using bacterial cells of *E. amylovora* (serial 3-fold dilutions of samples from 1 × 10^8^ to 500 CFU/mL) instead of the dsDNA cis-target.

### 2.9. Cas12a-Based Assay of E. amylovora Combined with RPA

RPA was performed using the RPA basic kit (TwistDx, Cambridge, UK) according to [32]. Briefly, labeled primers were mixed with the rehydration buffer to a final concentration of 300 nM. Next, 10 µL of the sample with bacterial cells of *E. amylovora* was added from a range of 1 × 10^7^ to 500 CFU/mL. The lyophilized pellet (basic RPA kit) was dissolved into the mixture. Further, Mg(CH_3_COO)_2_ was added to a final concentration of 14 mM; the reaction was performed for 20 min at 37 °C in the T100 Thermal Cycler (BioRad, Hercules, CA, USA). After the reaction, 3 µL of the RPA mixture solution was inserted into the Cas12a–gRNA mixture with the probe. A Cas12a-based assay was performed, as described before (see Section 2.6).

## 3. Results and Discussion

### 3.1. Design of Experiments

To search optimal structures of ssDNA probes with anchors, we operated with the following assumptions: (1) the anchor should be an easily accessible compound, (2) it should be readily incorporated into the ssDNA probe, (3) it should provide a significant difference in FA before and after cleavage, and (4) it should not hinder cleavage of the ssDNA by Cas12a. Accordingly, we proposed two approaches. The first approach involved using a protein as an anchor. For this approach, streptavidin (STR) was selected, which can be readily introduced through the biotin label on the ssDNA probe. The streptavidin–biotin interaction is characterized by extremely high affinity, selectivity, and stability of the formed complexes. These properties and easy biotinylation of biomolecules ensure successful application of the STR–biotin complexes in biosensors for the detection of biomarkers, such as proteins and nucleic acids [33,34,35]. Moreover, STR has already proven itself as an anchor, for example, in aptamer-based biosensors [20]. To implement this approach, we used ssDNA (both linear structures (6 types) and hairpin structures (4 types) of different compositions), with a fluorescent label (FAM) at one end and biotin at the opposite end (the structures are presented in Table 1, lines 1–10). A simple addition of STR to the probe formed a robust anchor structure, ensuring high FA. After the cleavage of the ssDNA by activated Cas12a, FAM and STR were not connected in a single structure, leading to a decrease in FA.

The second approach involved using the ssDNA itself as the anchor. For these anchors, we selected a G-quadruplex as a highly organized structure, a hairpin structure with a long loop part (21 nt), and a long linear sequence (30 nt) that, due to its length, could provide a sufficiently significant increase in FA compared to probes of 8–15 nt length. For linear probes, it has been shown that the cleavage efficiency does not change starting from a length of 8 nt [36]. To implement this approach, only one label, FAM, at one end of the ssDNA was sufficient. All the structures are presented in Table 1, lines 11–13.

Additionally, we hypothesized that the use of divalent metal salts would increase the FA of uncleaved structures based on data suggesting an increase in the torsion and rigidity of nucleic acids in the presence of divalent metal ions [30,31]. Therefore, we thoroughly investigated the influence of Mg^2+^, Ca^2+^, and Ba^2+^ salts at different concentrations on the change in FA before and after cleavage, using linear probes as an example. All salts were added after the trans-cleavage reaction (see Section 2.6 for details), so the optimal buffer for measuring the FA of probes may differ from the optimal buffer for the Cas12a reaction and is easy to implement in practice.

**Figure 1:**
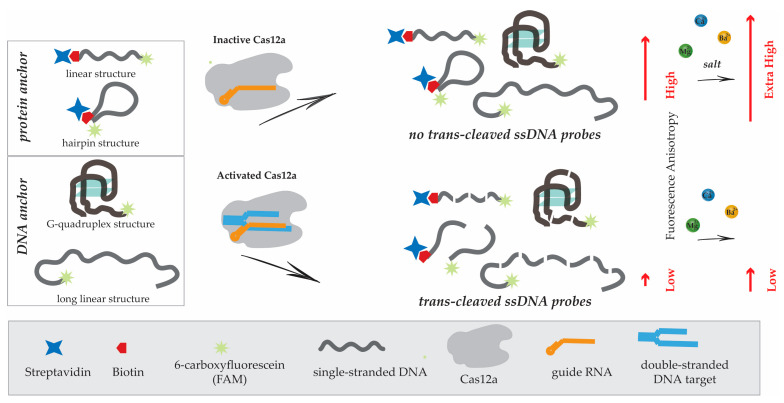
Schematic of FA assay based on CRISPR/Cas12 using different ssDNA probes with anchors.

In the assay using the provided probes, the complex of Cas12a with gRNA recognizes the dsDNA cis-target, which triggers the activation of Cas12a’s trans-nuclease activity. The trans-nuclease activity of Cas12a leads to the cleavage of the ssDNA probe, resulting in decreasing FA. In the absence of the dsDNA cis-target, Cas12a activation does not occur, and the FA signals of the probes remain high. The schemes of the FA assays based on CRISPR/Cas12 using different ssDNA probes with anchors are presented in Figure 1.

As the cis-target DNA, we used a gene fragment of *E amylovora* (phytopathogen that causes fire blight [37]), which enables a specific diagnosis in a CRISPR/Cas12a-based assay with RPA [32].

### 3.2. Protein Anchor, Its Impact on Fluorescent Anisotropy (FA), and Strategies to Increase FA

**Figure 2:**
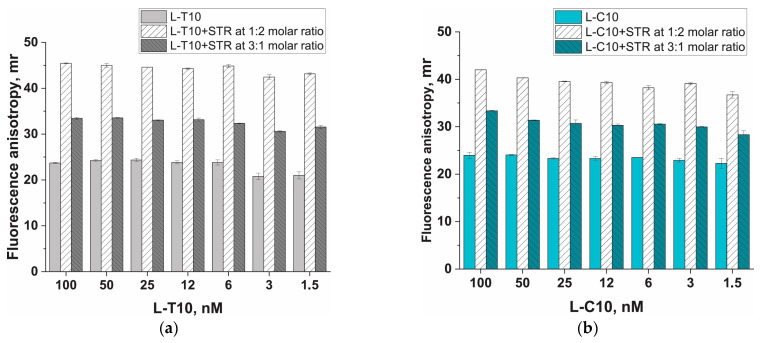
Dependence of fluorescence anisotropy on the ssDNA concentration in the absence of streptavidin (STR), as well as at molar ratios of probe:STR of 1:2 and 3:1, obtained for L-T10 (**a**) and L-C10 (**b**) probes. Experiments were carried out in 20 mM Tris-HCl buffer, pH 9. Each experiment was repeated in triplicate.

To verify the basic hypothesis regarding the influence of a protein anchor on increasing FA, we evaluated the FA of probes without an anchor and with it at different concentrations. The selected protein anchor, STR, is capable of simultaneously binding up to four biotin molecules [38]. Consequently, depending on the conditions, streptavidin-(biotinylated probe) conjugates can be obtained in molar ratios ranging from 4:1 to 1:1. We selected linear oligonucleotides labeled with both FAM and biotin, L-T10 and L-C10 (see Table 1), and mixed them with streptavidin in a 3:1 molar ratio (to obtain multiple probes on a single streptavidin anchor) and a 1:2 molar ratio (with each probe having an individual streptavidin anchor). The results of FA measurements for different concentrations of the probe are presented in Figure 2 (fluorescence data are provided in Supplementary Materials, Figure S1), allowing several conclusions to be drawn. First, there was a distinct difference in FA between the probe with an anchor and the probe without an anchor. Second, in the range of probe concentrations from 1 to 100 nM, FA remained stable regardless of the presence of an anchor (while the fluorescence signal increased with increasing concentration; see Figure S1). Third, for both probes, the greatest FA difference between the probe with and the probe without an anchor was shown for the 1 (probe):2 (STR) molar ratio. Fourth, for the L-T10 and L-C10 structures, the maximum difference in FA between the probe with an anchor and the probe without one varied from 18 to 22 mr. These ∆FA values were significant distinctions but not high enough for a quantitative assessment of the ssDNA probe cleavage process in a Cas12a-based assay.

The obtained results indicate that in the presence of ssDNA connecting STR and fluorescein, FA does not increase as significantly as it was shown in other studies using an STR anchor [20,39]. Presumably, ssDNA has the flexibility to allow the intramolecular rotation of fluorescein and strong light depolarization. Upon probe hydrolysis, ΔFA is considered between the non-cleaved probe and the fluorescein attached to the cleaved probe fragment (~3 mr). Therefore, the obtained ΔFA of approximately 40 mr (maximum-possible ΔFA is approximately 400 mr [19]) appears to be insufficient for constructing calibration dependencies. In this situation, the following several strategies can be proposed to make the protein anchor more effective.

The first strategy involves increasing the rigidity of the connection between STR and fluorescein. This can be achieved by adding an ssDNA sequence complementary to the ssDNA probe after its cleavage by Cas12a. Probes that are not cleaved will form double-stranded structures with greater rigidity and a higher FA signal, as demonstrated in several studies [40,41]. We tested this strategy by adding a complementary oligonucleotide, A10, to STR–L-T10. However, the addition did not affect the FA increase, whether added to the probe itself or to the probe with STR (results are presented in Supplementary Materials, Figure S2).

The second strategy assumes that the addition of divalent metal ions to the buffer used for FA measurement will lead to greater compaction of the ssDNA probe, as supported by several studies [42,43,44], and consequently to FA increase. This assumption was also supported by the results obtained from the first strategy, where complementary interactions were conducted in a buffer containing 10 mM Mg^2+^, unlike the experiment presented in Figure 1. Thus, the addition of 10 mM Mg^2+^ resulted in an increase in FA by 32.2 mr for L-T10 and 60.9 mr for STR-L-T10. Accordingly, the difference between the anchorless probe and the anchored one was 50.5 mr (Supplementary Materials, Figure S2), which corresponded to an increase of approximately 130% (from 21.6 to 50.5 mr). A detailed investigation of the influence of divalent metals is discussed in Section 3.3.

Finally, the third strategy is based on bringing STR and fluorescein closer together, thereby reducing the mobility of fluorescein relative to the anchor. This approach can be implemented by decreasing the number of nucleotides in the ssDNA part, but it is limited to the minimum number required for the Cas12a cleavage (5 nt according to [4,5,6]). Alternatively, proximity can be achieved by using a hairpin structure, in which STR and fluorescein are close at the ends of the stem, and the loop will be cleaved by Cas12a and can be of the appropriate length. We proposed variants of such structures (see lines 1–13 in Table 1). A detailed investigation of the influence of the probe structure on the FA signal is discussed in Section 3.4.

### 3.3. Divalent Metal Ions and Their Influence on Fluorescent Anisotropy

**Figure 3:**
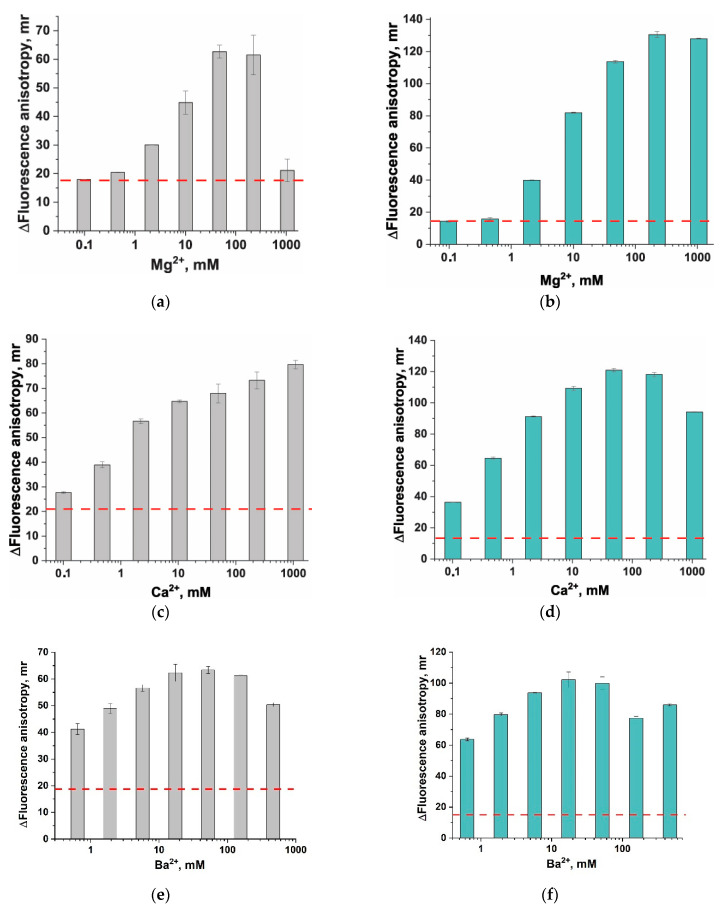
Impact of divalent metal ions on fluorescence anisotropy (FA) of a probe and a probe with STR (**a**,**c**,**e**) for L-T10 and (**b**,**d**,**f**) for L-C10. The difference in the FA of the SRT–probe and the probe on the concentration of Mg^2+^ (**a**,**b**), Ca^2+^ (**c**,**d**), and Ba^2+^ (**e**,**f**). The dashed lines indicate ∆FA in the absence of Me^2+^. Each experiment was repeated in triplicate.

For testing the second strategy, we chose several divalent metal cations: Mg^2+^, Ca^2+^, Ba^2+^, and Zn^2+^. Divalent cations salts were added to the linear probes L-T10 and L-C10, both in the absence of an anchor and in complex with the anchor in the optimal molar ratio (1 (probe):2 (STR)). The concentrations of the metal salts covered the range of cations from 1000 to 0.1 mM (for Zn^2+^ to 1 µM). For all examined metal ions, an increase in their concentration led to an increase in the FA of both conjugates of L-T10 and L-C10 with STR and for the ssDNA probes themselves (Supplementary Materials, Figure S3), accompanied by a decrease in the intensity of fluorescence (Supplementary Materials, Figure S4). In the presence of Mg^2+^, Ca^2+^, and Ba^2+^, similar behavior of FA was observed: a significant increase over the entire concentration range with the maxima ranging from 50 to 200 mM for Mg^2+^ and Ca^2+^ and from 20 to 50 mM for Ba^2+^ (Figure 3). In all cases, this difference was higher for L-C10 (see Figure 3a,c,e) than for L-T10 (see Figure 3b,d,f), even though in the absence of divalent metal ions, ΔFA was higher for L-T10 (see dashed lines in Figure 3). In the presence of Zn^2+^, a sharp increase in FA was noted up to 0.1 mM, followed by a sharp decrease at higher concentrations (Supplementary Materials, Figure S5). The expected precipitation of complexes with zinc ions under the selected conditions was observed. Despite the fact that zinc ions provide the greatest difference between the initial probe and the anchored probe (~170 mr for L-C10), their application is problematic from a practical point of view and requires more fine-tuned conditions due to the tendency to precipitate. The most interesting variant is the use of 50–200 mM Mg^2+^ and Ca^2+^ solutions, as they provide ΔFA = 120 mr in the case of the L-C10 probe and do not show precipitation. Thus, this variant demonstrated high efficiency, increasing ΔFA by ~670% (from 18 to 120 mr).

Additionally, we assessed the effect of the anchor on L-A10 at different concentrations of Mg^2+^, Ca^2+^, and Ba^2+^ (results are given in Supplementary Materials, Figures S3, S4, and S6) and found that ΔFA of L-A10 with and without an anchor is lower than for L-C10.

The reasons for such a significant effect are primarily related to the nature of nucleic acids. Nucleic acids are polyanions that need to be shielded to enable the formation of a stable secondary structure, so they naturally interact with different cations in solution. Metal ions directly influence the stability and spatial conformation of DNA and RNA molecules primarily through non-specific interactions governed by electrostatic forces [30,31]. The metal ions increase the compaction of ssDNA by reducing repulsion between links of the same strand along the negatively charged backbone. As evidence for this explanation, the data obtained by Chen et al. [42] showed a decrease in the end-to-end distance for dT40 and rU40, labeled by Cy3 and Cy5 at opposite ends of oligonucleotides in the presence of MgCl_2_ (50 mM) or even NaCl (3000–6000 mM). The distance between the opposite ends under the same concentration of metal ion depends on the nucleic acid composition. The probable cause of the increased FA of the ssDNA probe without an anchor is that divalent metal ions make the fluorescence-labeled oligonucleotide stiffer, restraining the fluorophore’s mobility at its end. Less mobility of the oligonucleotide leads to higher FA. A similar effect of increasing FA was demonstrated by Ellouze et al. when comparing poly(dT) and poly(dA) duplex and triplex formation under different magnesium ion concentrations [43].

### 3.4. Selection of the ssDNA Probe Structure to Maximize Fluorescent Anisotropy in Combination with a Protein Anchor

**Figure 4:**
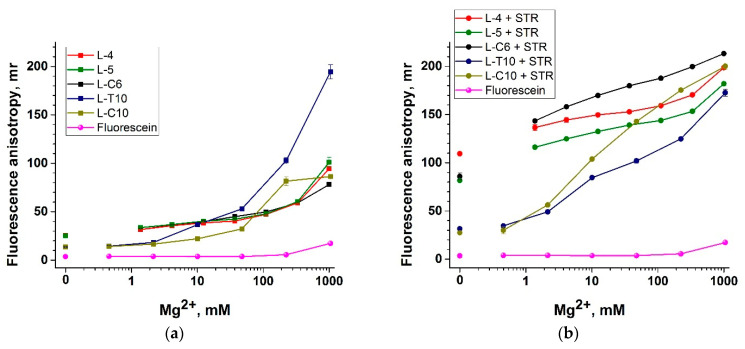

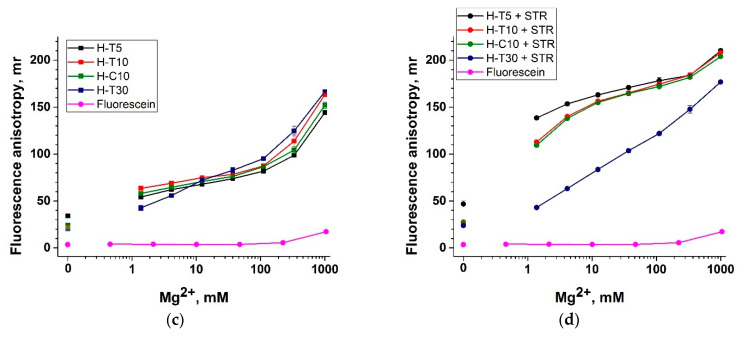
Impact of divalent metal ions on fluorescence anisotropy (FA) of FAM-labeled linear (**a**,**b**) and hairpin (**c**,**d**) structures of probes. Dependence of the FA of FAM-labeled probes on the concentration of Mg^2+^. Each experiment was repeated in triplicate.

The influence of the ssDNA structure on the FA of fluorescein was estimated in the presence of Mg^2+^ salts. In all experiments, we also monitored the FA of fluorescein itself. First, these data allowed us to control for artifacts at high salt concentrations, and second, fluorescein serves as an absolute reference for FA in the case of 100% probe cleavage by Cas12a. We compared five linear structures (see lines 1–5 in Table 1), including the previously used L-T10 and L-C10, and structures with fewer nucleotides between FAM and STR (Figure 4a,b). The choice of polyC and polyT sequences was primarily related to their cleavage efficiency by Cas12a. The cleavage efficiency increases in the row polyT < polyA < polyC [36,45]. PolyG and G-rich reporters demonstrate either low cleavage or its absence [36,45].

The L-4, L-5, and L-C6 probes demonstrated similar dependence of FA on the Mg^2+^ concentration in the absence of an anchor and in the presence of an anchor, showing significant differences in FA in the salt concentration range from 1 to 1000 mM. Moreover, even in the absence of divalent metal ions, the FA of shortened probes with an anchor was high (80–110 mr) (see Figure 4b). A similar FA was a characteristic of L-C10 at high Mg^2+^ concentrations (~100 mM). Thus, the obtained results demonstrate that bringing FAM and STR closer together by shortening the ssDNA probe is an effective strategy for FA increase. However, L-C10 provides better accessibility of nucleotides for cleavage by Cas12a and was selected, assuming that the FA of L-C10-STR at 100 mM Mg^2+^ was close to that of short probes.

The second group for comparison included four hairpin structures (see lines 7–10 in Table 1), among which were H-T10 and H-C10, having the same nucleotides for cleavage in the loop as in L-10T and L-C10. The group also included shorter (5 nucleotides) and longer (30 nucleotides) structures. For these structures (H-T5, H-T10, H-C10, H-T30), we used the stem, the efficiency of which has been previously demonstrated in experiments on trans-cleavage with Cas12a [46]. Rossetti and co-authors first showed that hairpin probes are more efficient than linear ones for trans-cleavage of Cas12a [46]. All probes without an anchor had higher FA values (near 20 mr; see Figure 4a,c) than the previously examined linear structures. Three hairpin probes (H-T5, H-T10, H-C10) with an anchor had FA values that were close to each other and to the FA of short linear conjugates, especially in the high salt range. Meanwhile, H-T30 with an anchor showed similarity to L-T10. Thus, hairpin structures with an anchor also showed improvements only in low-salt solutions, and at high salt concentrations, there was no significant improvement in FA. However, according to [46], the hairpin structure provides better conditions for cleavage by Cas12a. As a result of the comparative analysis, we selected two homologous oligonucleotides to assess the sensitivity of the probes in a Cas12a-based assay: L-C10 and H-C10 (see Section 3.6).

### 3.5. Nucleic Acid Anchors and Its Impact on Fluorescent Anisotropy

The probes without an anchor in conditions of high concentrations of divalent metal ions showed high FA (see Figure 4a,c). These results demonstrate that the nucleic acid itself serves as a sufficient anchor. This was additionally confirmed by the comparison of the FA of probes with the FA of fluorescein, which is a potential signal when the DNA probe is fully cleaved by Cas12a. To test this statement, we decided to study three probes (see lines 11–13 in Table 1) with the FAM label:

(1) G-quadruplex with an extended end on the 3′-end ending with fluorescein (G-quadruplex). We used an aptamer specific to ochratoxin A (OTA) [47] since it has a tightly organized structure, which increases the anisotropy of labeled OTA.
(2) A long (30 random nucleotides) DNA oligonucleotide that does not form any stems or other secondary structures (L-R30).
(3) A 37-nucleotide hairpin, including 21 random nucleotides in the loop.

**Figure 5:**
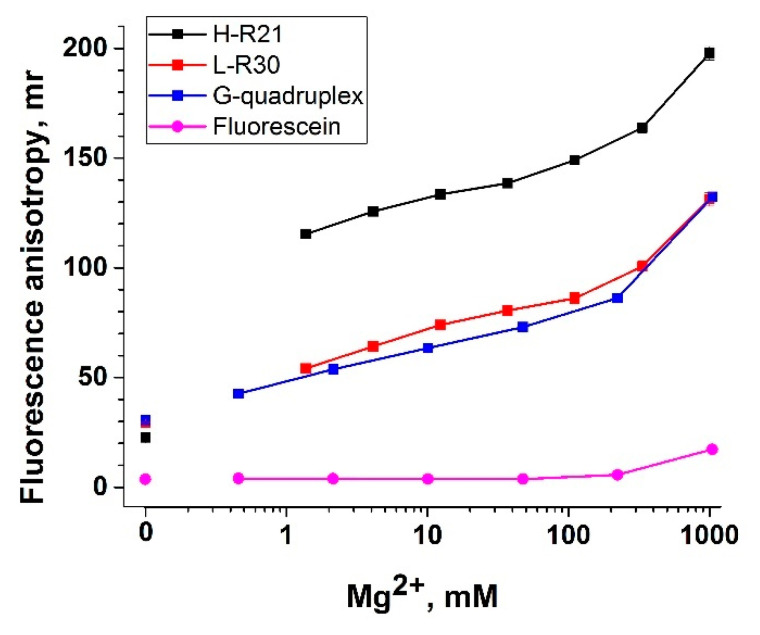
Impact of divalent metal ions on fluorescence anisotropy (FA) of FAM-labeled G-quadruplex, L-R30, and H-R21 probes. Dependence of the FA of FAM-labeled probes on the concentration of Mg^2+^. Each experiment was repeated in triplicate.

Studying these probes, we measured the FA at different amounts of Mg^2+^. The obtained results are shown in Figure 5. The anisotropy of the 3′-end fluorophore in the G-quadruplex (40 nucleotides) demonstrated similar dependence with the linear structure L-R30 (30 nucleotides). To explain this similarity, we characterized the G-quadruplex with circular dichroism (CD) in the absence of divalent metal salts, at 10 and 100 mM Mg^2+^ (all variants in 50 mM Tris-HCl buffer, pH 9). The CD spectra showed that the G-quadruplex structure was formed only for the experiment with 10 mM Mg^2+^; in other cases, the characteristic peaks of the G-quadruplex (max. at 246 and 292 nm, min. at 236 and 261 nm) were absent (Supplementary Materials, Figure S7). Regardless of whether the G-quadruplex was formed, the linear and G-quadruplex structures induced the same FA under the considered salt conditions (from 1 to 1000 mM Mg^2+^). Thus, FA was determined by the length of the nucleotide chain and the salt composition for the structures considered. At the same time, in Figure 5, there is a significant (~50 mr) difference between H-R21 and L-R30 and the G-quadruplex, which is manifested only in the presence of divalent metal ions. The FA values for H-R21 were comparable to the FA of the protein anchor (see Figure 4b,d). Note that the FA of hairpin probes (H-T5, H-T10, H-C10, H-T30) without anchors was significantly lower. Based on the results obtained in this section, H-R21 and the G-quadruplex were selected for assessing the sensitivity of the probes in a Cas12a-based assay (see Section 3.6).

### 3.6. Comparison of Probes in Cas12a-Based Assay

Based on the previous results, we selected four probes with anchors for Cas12-based assays with FA detection: L-C10 and H-C10 with a protein anchor and H-R21 and the G-quadruplex with a nucleic acid anchor. We used the CRISPR/Cas12a recognition system with gRNA (the sequence is provided in Supplementary Materials, Table S1), specifically recognizing a 21-nucleotide fragment of the AMY1267 gene of *E. amylovora* [32]. The dsDNA with a length of 157 bp containing the target fragment was generated using PCR and purified (the method and characteristics proving homogeneity of the obtained product can be found in Section 2.5 and Supplementary Materials, Figure S8).

For the initial assessment of the anchor’s impact on ssDNA cleavage by activated Cas12a, we used 0.3 and 10 nM of dsDNA, as well as a negative control that did not contain the target dsDNA. The Cas12a–gRNA complex was combined with dsDNA or a negative control simultaneously with the probe. The probes tested included L-C10 without an anchor, L-C10 with the STR anchor (1:2) added to the reaction after the trans-cleavage reaction, and the L-C10–STR (1:2) conjugate obtained before the trans-cleavage reaction. After 30 min, the reaction mixture was adjusted to the optimal salt conditions: 50 mM Tris-HCl and 50 mM CaCl_2_, or 50 mM Tris-HCl and 100 mM Mg(CH_3_COO)_2_.

**Figure 6:**
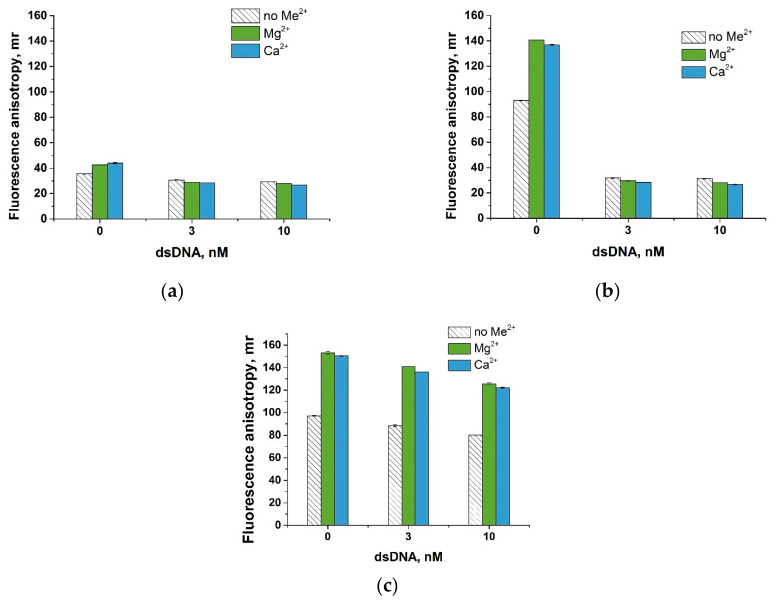
Comparison of ssDNA probes to detect the target dsDNA using CRISPR/Cas12 assay. Dependence of fluorescence anisotropy on the concentration of the target dsDNA obtained for L-C10 (**a**) and L-C10 and STR (1:2) added to reaction after trans-cleavage (**b**) and the L-C10–STR (1:2) conjugate obtained before the trans-cleavage reaction (**c**). Each experiment was repeated in triplicate.

The results showed that in the absence of an anchor, the change in FA was negligible compared to the negative control and FA was low for three variants (Figure 6a). When using the STR anchor, the results depended on the method of forming the STR–L-C10 conjugate. When forming the conjugate before trans-cleavage, STR interfered with cleavage and the change in FA was negligible compared to the negative control, with high FA for three variants (Figure 6c). When forming the conjugate after trans-cleavage, STR did not hinder cleavage and the FA signal significantly decreased compared to the negative control (Figure 6b). These results also confirm that the conclusions made regarding the impact of divalent metals on enhancing the FA of the probes are valid in the CRISPR/Cas12a system. Given that the differences between enhancements in the presence of Mg^2+^ and Ca^2+^ are insignificant, we chose 100 mM Mg^2+^, the salts of which are present in the optimal buffer for CRISPR/Cas12a (10 mM Mg^2+^).

In the hairpin structure H-C10, in contrast to the linear L-C10, streptavidin was located as close as possible to fluorescein, and at the same time, it was outside the cutting site (loop). Due to this design, streptavidin did not interfere with the trans-cleavage of H-C10 and could be assembled prior to cleavage of the probe.

For the four ssDNA probes selected for analysis (G-quadruplex, L -C10, H-R21, H-C10), polyacrylamide gel electrophoresis was performed under denaturing conditions to confirm trans-cleavage only in the presence of the dsDNA target. Accordingly, we carried out the Cas12a-based assay in the presence of 10 nM of dsDNA and without it for the probes G-quadruplex, L-C10 + STR after assay, H-R21, and H-C10 + STR before assay. We used the G-quadruplex, L-C10, H-R21, and H-C10 probes as controls. We detected the position of the non-cleaved probes by the fluorescence of the fluorescein label at the end of the probes. As a result, four control probes were visualized according to the lengths of the probes (see the scan of the gel in Supplementary Materials, Figure S9). For all samples where the dsDNA target was present in the assay, no colored bands were observed, which indicates complete trans-cleavage of the probe; fluorescein with residual nucleotides left the gel much earlier than the probes (see Figure S9). For all samples without dsDNA, staining of non-cleaved probes was observed (see Figure S9). Moreover, the positions of the G-quadruplex and H-R21 corresponded to the positions of these probes in the control samples, and the positions of L-C10 + STR and H-C10 + STR were much higher, because both of these probes are bound to the STR anchor.

**Figure 7:**
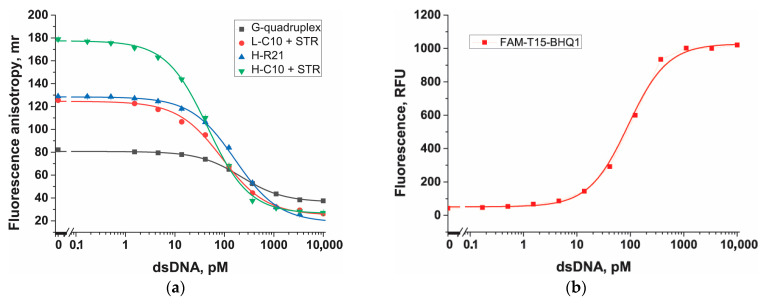

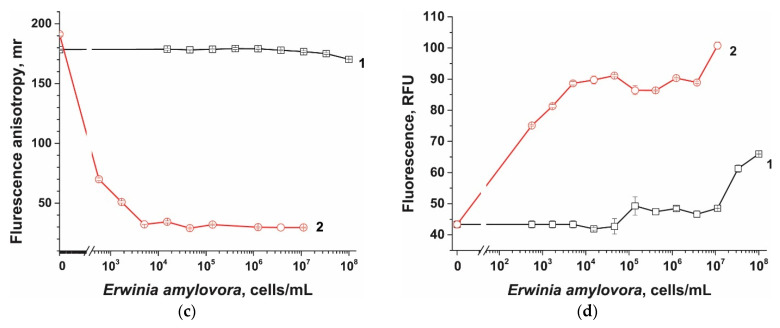
Concentration dependence for dsDNA (**a**,**b**) and bacterial cells (**c**,**d**) obtained using fluorescence anisotropy (**a**,**c**) and fluorescence (**b**,**d**). The probes were used at a 100 nM concentration. Streptavidin at a concentration of 200 nM was added after trans-cleavage for L-C10 and before for H-C10. 1 corresponds to a Cas12-based assay without RPA, and 2 corresponds to a Cas12-based assay combined with RPA. Each experiment was repeated in triplicate.

In the next stage of the FA probe testing, calibration curves of FA on dsDNA (in the concentration range from 0.2 pM to 10 nM) were obtained for the Cas12a-based assay with FA detection (Figure 7a) and with fluorescent detection (Figure 7b). Fluorescent detection was used to compare the sensitivity of the FA probes, with a typical probe labeled with fluorescein and a quencher (FAM-T15-BHQ1). Figure 7a shows sigmoidal curves for FA probes with the following limits of dsDNA detection: 0.8 ± 0.3 pM (H-C10 + STR before the trans-cleavage reaction), 2.0 ± 0.4 pM (L-C10 + STR after the trans-cleavage reaction), 5.1 ± 0.4 pM (H-R21), and 16.1 ± 1.3 pM (G-quadruplex) (all fitting parameters, LOD, and LOQ are given in Table 2). The detection limit with the FAM-T15-BHQ1 probe was 6.7 ± 1.7 pM (fitting parameters, LOD, and LOQ are given in Table 2). Therefore, all FA probes with anchors exhibited high sensitivity under the selected conditions (50 mM Tris-HCl, 100 mM Mg(CH_3_COO)_2_, pH 9.0).

The H-C10–STR probe offers several advantages over the only known FA detection system in Cas12a-based assays [29]: (1) H-C10-STR is a pre-made structure that can be easily obtained in advance, making it simpler than adding a DNA nanosheet after the trans-cleavage reaction, (2) STR is a component that does not require special synthesis, and (3) the detection limit for the cis-target is almost 50 times lower than that for FA based on DNA nanosheets.

The H-C10–STR probe was tested for the detection of *E. amylovora* bacteria in the samples using two methods. The first method involved adding bacterial cells at different concentrations (after heating at 95 °C for 10 min) to the CRISPR/Cas12a system instead of dsDNA targets. This approach showed low sensitivity (1 × 10^7^ cells/mL or 1 × 10^5^ cells per reaction) for both FA and the fluorescent probe (Figure 7c,d), which is consistent with the known low sensitivity of CRISPR/Cas12a without additional amplification methods [48]. The second method included performing prior RPA for bacterial cells at different concentrations, followed by the CRISPR/Cas12a reaction. In the case of RPA–CRISPR/Cas12a reactions, both the FA detection probe and the fluorescent detection probe exhibited equally high sensitivity (approximately 500 cells/mL or 5 cells per reaction) (Figure 7c,d). Furthermore, the FA probe showed better reproducibility of results upon repeat testing (Figure 7c,d). The better reproducibility and signal stability in various environments affecting fluorescence (e.g., salt solutions or multi-component matrices that may quench fluorescence) support the fact that an anchor-based probe is a promising tool for further use in CRISPR/Cas12a systems.

## 4. Conclusions

The important feature of Cas12a is its trans-nuclease activity against any ssDNA, acquired after successful cis-target DNA recognition. Among the methods for detecting the trans-cleavage of ssDNA probes, FA has been underused. This could be due to the requirement of developing new probes with different FA values before and after cleavage. However, by applying the concept of anchor-based enhancement and exploring the potential of divalent metal ions to increase FA, we identified several effective universal probes.

We investigated anchor-based enhancement using streptavidin, the G-quadruplex, linear, and hairpin nucleotide structures. For each of these anchors, we demonstrated the FA increase in the presence of the anchor. Additionally, we showed for the first time the dependence of the FA of various DNA probes on the concentration of divalent ions and how this property can be leveraged in Cas12a-based assays.

The comparative analysis of several anchor-based probes under optimal salt conditions revealed that the highest sensitivity, which was eight times stronger than that of a conventional fluorophore-quencher probe, was achieved with the hairpin structure, including 10 dC in the loop and streptavidin, located close to fluorescein, at opposite ends of the stem. This structure enabled the detection of the dsDNA cis-target in the Cas12a-based assay with a detection limit of 0.8 ± 0.3 pM and the detection of *E. amylovora* cells in an RPA-Cas12a-based assay with a detection limit of approximately 500 cells/mL.

These results suggest that Cas12a-based assays with FA detection may be useful in diagnostics. First, FA offers advantages in signal stability and reproducibility compared to fluorescence-quenching components. Second, the universal and simple FA probes developed in this study can be applied in any Cas12a-based assay.

## Figures and Tables

**Table 1:** Sequences and structures of ssDNA probes used in this study.

№.	Short Name	Sequence 5′-3′	Structure and Purpose
1	L-4	FAM-TTAT-Bio	Linear structure for protein anchor
2	L-5	FAM-TTATT-Bio	
3	L-C6	FAM-CCCCCC-Bio	
4	L-T10	FAM-TTTTTTTTTT-Bio	
5	L-C10	FAM-CCCCCCCCCC-Bio	
6	L-A10	FAM-AAAAAAAAA-Bio	
7	H-T5	FAM-**CTCTC**ATTTTTA**GAGAG**-Bio	Hairpin structure for protein anchor *
8	H-T10	FAM-**CTCTC**ATTTTTTTTTTA**GAGAG**-Bio	
9	H-C10	FAM-**CTCTC**ACCCCCCCCCCA**GAGAG**-Bio	
10	H-T30	FAM-**CTCTC**ATTTTTTTTTTTTTTTTTTTTTTTTTTTTTTA**GAGAG**-Bio	
11	H-R21	**AAAAAAA**CCTCCAAGAGTTAGATCATACA**GTTTTTTT**-FAM	Hairpin structure as DNA anchor
12	G-quadruplex	GATCGGGTGTGGGTGGCGTAAAGGGAGCATCGGACA-FAM	G-quadruplex structure as DNA anchor **
13	L-R30	FAM-GTTATTGCCGCCGCACAGCGTAAAGGTAAG	Linear structure as DNA anchor
14	A10	AAAAAAAAAA	Linear structure to form double strand with L-T10 probe
15	FAM-T15-BHQ1	FAM-TTTTTTTTTTTTTTT-BHQ1	Linear structure for fluorescence detection after trans-cleavage

Bio—biotin, FAM—fluorescein, BHQ1—black hole quencher1. * Sequences in bold form the hairpin stems. ** Sequence underlined forms a G-quadruplex.

**Table 2:** Fitting parameters for concentration dependence for dsDNA presented in Figure 7a,b and the calculated LOD and LOQ.

Probe/Parameter	H-C10 + STR	L-C10 + STR	H-R21	G-quadruplex	FAM-T15-BHQ1
A1	178.3 ± 0.9	125.0 ± 0.6	128.4 ± 0.5	81.0 ± 0.4	51.5 ± 1.0
A2	26.7 ± 0.4	25.4 ± 0.9	21.7 ± 0.8	37.0 ± 0.4	1028.1 ± 20.1
x0	44.5 ± 2.2	87.1 ± 5.3	150.0 ± 7.9	207.7 ± 9.9	91.1 ± 11.2
p	1.0 ± 0.1	1.0 ± 0.1	1.0 ± 0.1	1.0 ± 0.1	1.2 ± 0.1
IC10	5.3 ± 0.5	9.1 ± 1.1	17.9 ± 1.7	24.5 ± 2.6	14.2 ± 1.8
Reduced chi-sqr	54.1	11.1	7.21	1.2	38.4
Adj. R-square	0.9996	0.9989	0.9993	0.9990	1.0
LOD, pM	0.8 ± 0.3	2.0 ± 0.4	5.1 ± 0.4	16.1 ± 1.3	6.7 ± 1.7
LOQ, pM	1.4 ± 0.2	3.5 ± 0.3	3.5 ± 0.3	27.9 ± 1.2	10.5 ± 1.9

## Data Availability

The data presented in this study are available on request from the corresponding author.

## Supplementary Materials

The following supporting information can be downloaded at https://www.mdpi.com/article/10.3390/bios13121034/s1: Figure S1: Dependence of fluorescence on ssDNA concentration; Figure S2: Comparison of the fluorescence anisotropy of probes L-T10 and STR–L-T10 before and after interaction with A10; Figure S3: Fluorescence anisotropy of L-T10, L-C10, and their conjugates with a streptavidin anchor, depending on the concentration of Mg^2+^, Ca^2+^, Ba^2+^, and Zn^2+^; Figure S4: Fluorescence of L-T10, L-C10, and their conjugates with a streptavidin anchor, depending on the concentration of Mg^2+^, Ca^2+^, Ba^2+^, and Zn^2+^; Figure S5: Fluorescence anisotropy difference between the STR probe and the probe at different concentrations of Zn^2+^; Figure S6: Impact of divalent metal ions on the fluorescence anisotropy (FA) of the L-A10 probe and the probe with STR; Figure S7: CD spectra of the G-quadruplex; Figure S8: Scan of gel electrophoresis in 2% agarose gel of a fragment of the target AMY1267 gene of *Erwinia amylovora*; Figure S9: Scan of polyacrylamide gel after electrophoresis of ssDNA probes, mixtures after Cas12a-based assay with 10 nM of dsDNA and ssDNA probes, and mixtures after Cas12a-based assay without dsDNA and with ssDNA probes; Table S1: Sequences of primers and guide RNA used in this research.
