# Supplementary material for: DNA Probes for Cas12a-Based Assay with Fluorescence Anisotropy Enhanced Due to Anchors and Salts
Source: Biosensors (Basel). 2023 Dec 16;13(12):1034. doi: 10.3390/bios13121034 (PMC10741848; doi:10.3390/bios13121034)
Supplement: Supplementary file 1 [file biosensors-13-01034-s001.zip › biosensors-2734034-supplementary.pdf]

biosensors

Supplementary Material

DNA Probes for Cas12a-Based Assay with Fluorescence Anisotropy Enhanced due to Anchors and Salts

Irina V. Safenkova1, Alexey V. Samokhvalov1, Kseniya V. Serebrennikova1, Sergei A. Eremin1,2, Anatoly V. Zherdev1 and Boris B. Dzantiev1,*

1A.N. Bach Institute of Biochemistry, Research Centre of Biotechnology of the Russian Academy of Sciences, 119071 Moscow, Russia; safenkova@inbi.ras.ru (I.V.S.); a.samohvalov@fbras.ru (A.V.S.); ksenijasereb@mail.ru (K.V.S.), eremin-fbras@yandex.ru (S.A.E); zherdev@inbi.ras.ru (A.V.Z.); dzantiev@inbi.ras.ru (B.B.D.)
2Faculty of Chemistry, M.V. Lomonosov Moscow State University, Moscow 119991, Russia
* Correspondence: dzantiev@inbi.ras.ru; Tel.: +7-495-954-2804

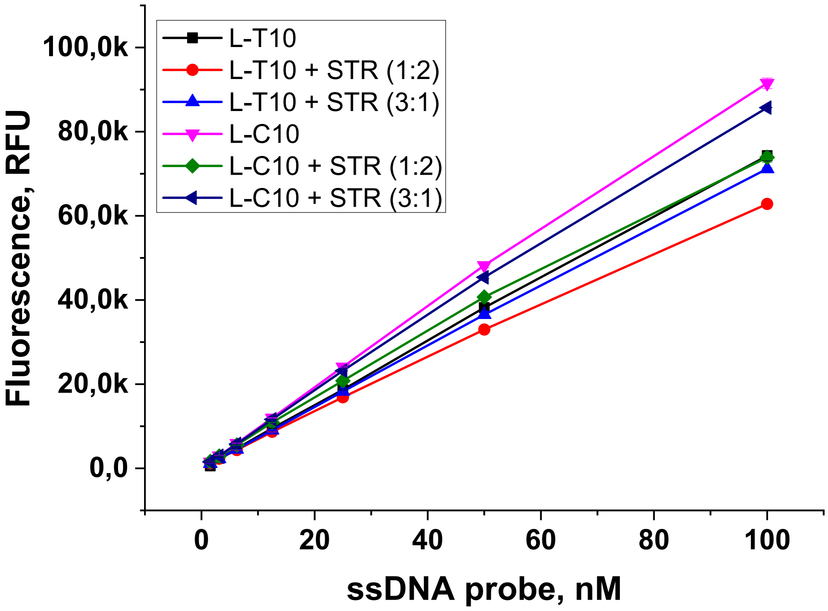

Figure S1. Dependences of fluorescence on ssDNA concentration in the absence of streptavidin (STR), as well as at molar ratios of probe : STR of 1:2 and 3:1. Experiments were carried out in 20mM Tris-HCl buffer, pH 9.

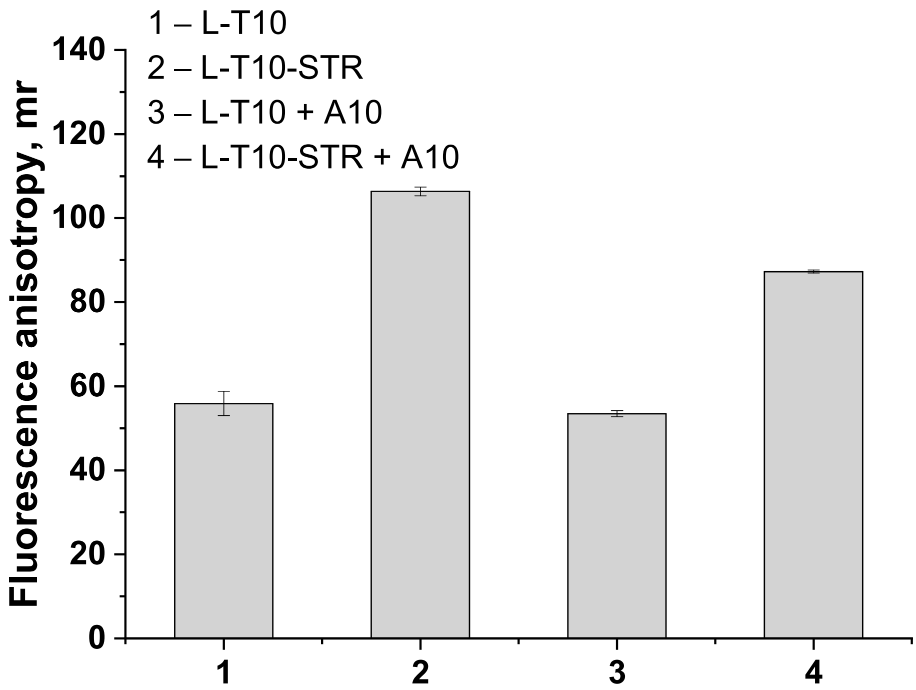

Figure S2. Comparison of the fluorescence anisotropy of probe L-T10 and STR – L-T10 before and after interaction with A10. Experiments were carried out in 20 mM Tris-HCl buffer containing 10 mM Mg^{2+}, pH 9.

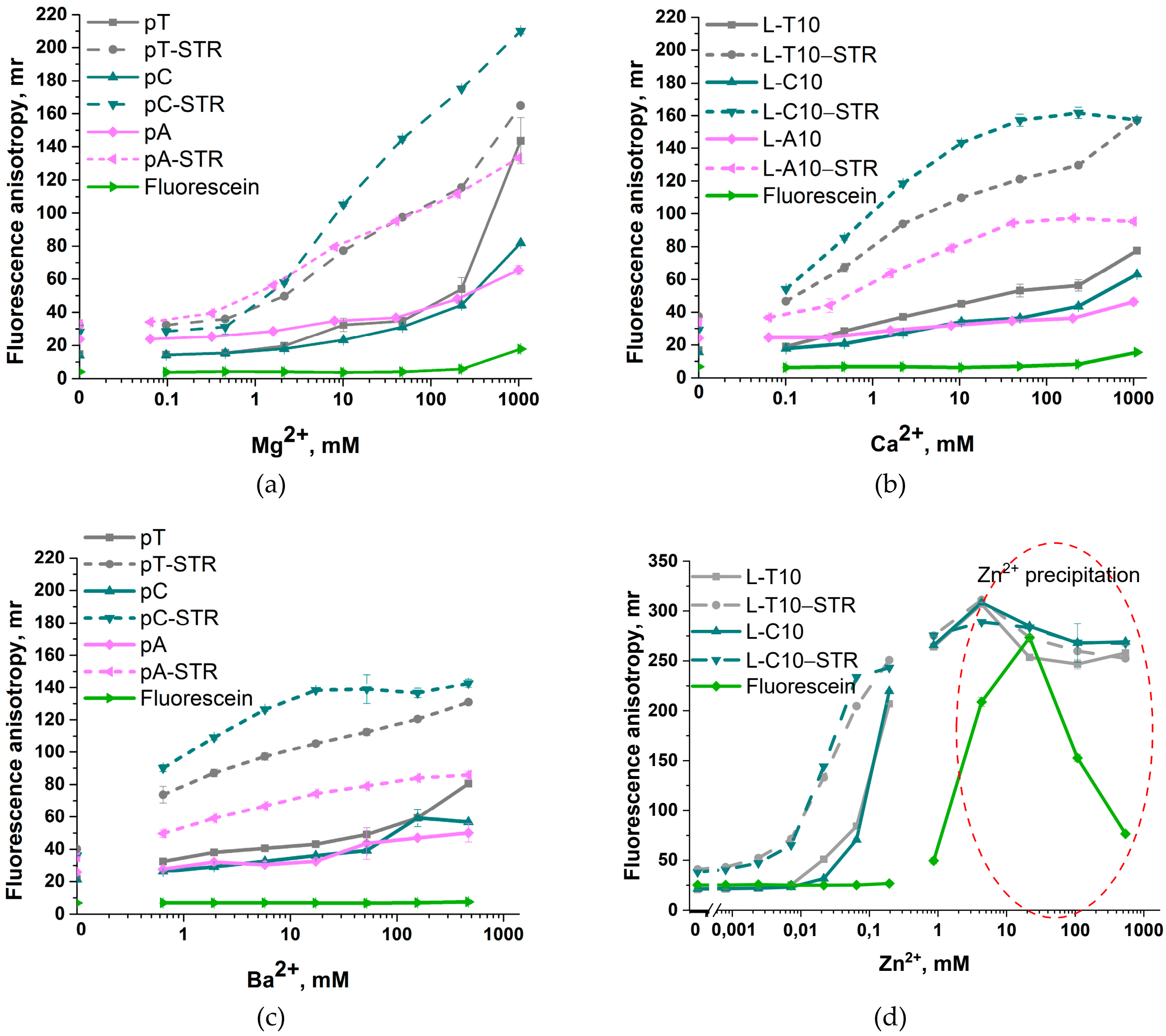

Figure S3. Fluorescence anisotropy of L-T10, L-C10, L-A10 and their conjugates with streptavidin anchor at 1:2 molar ratio depending on the concentration of Mg^{2+} (a), Ca^{2+} (b), Ba^{2+} (c), and Zn^{2+} (d) in 50mM Tris-HCl, pH 9.

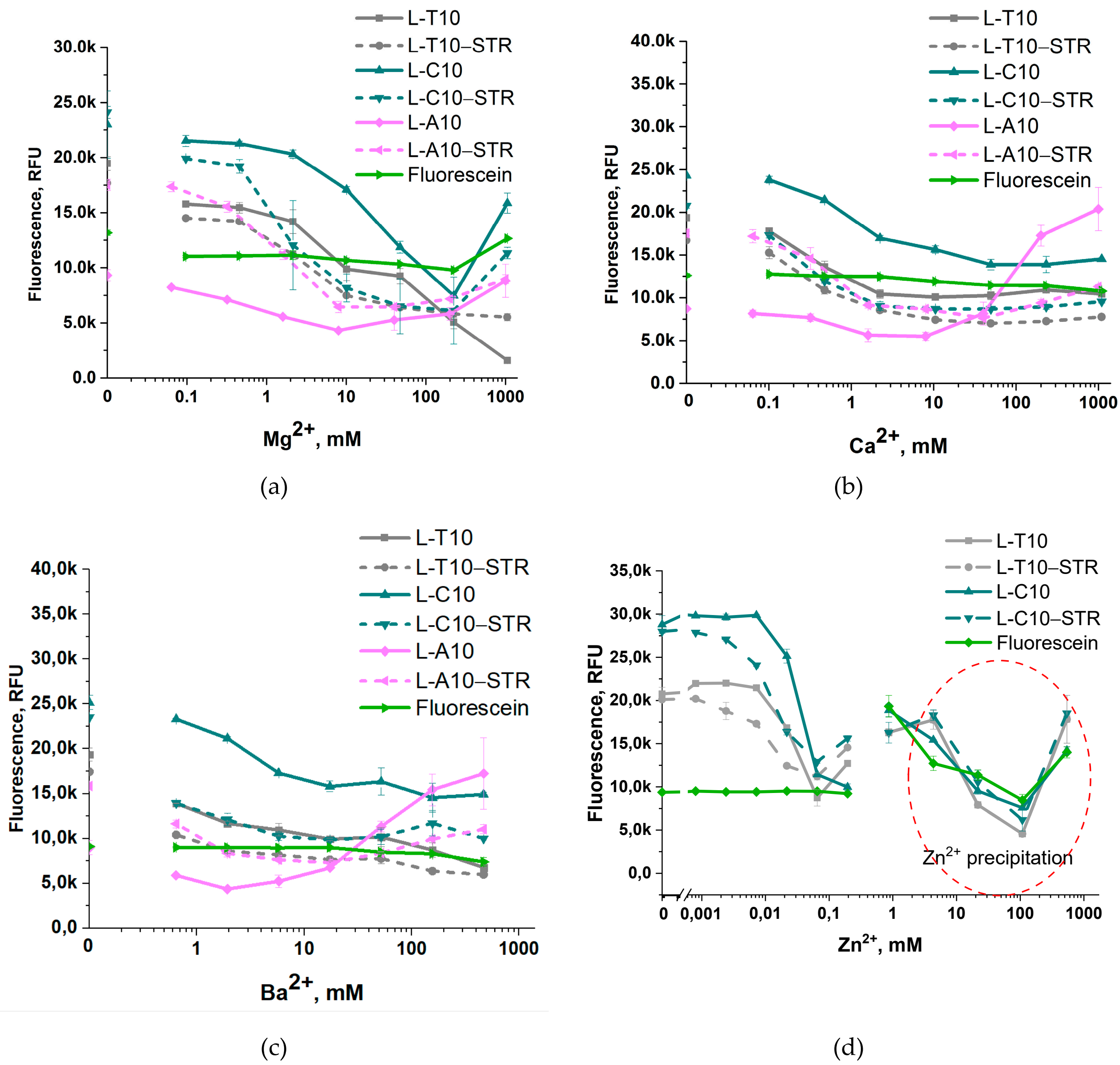

Figure S4. Fluorescence of L-T10, L-C10, L-A10 and their conjugates with streptavidin anchor at 1:2 molar ratio depending on the concentration of Mg^{2+} (a), Ca^{2+} (b), Ba^{2+} (c), and Zn^{2+} (d) in 50mM Tris-HCl, pH 9.

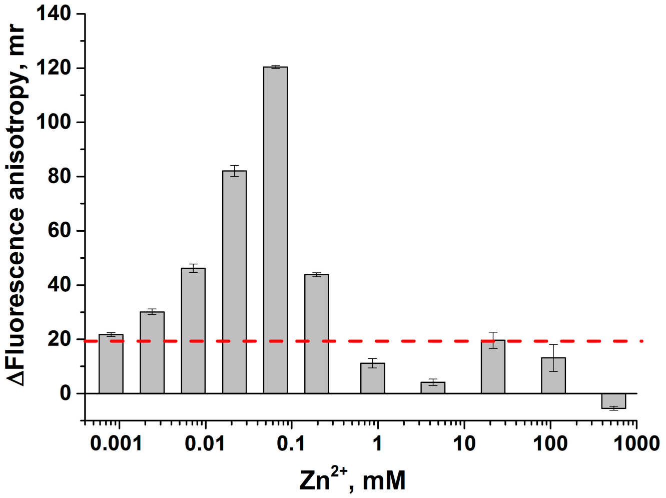

a

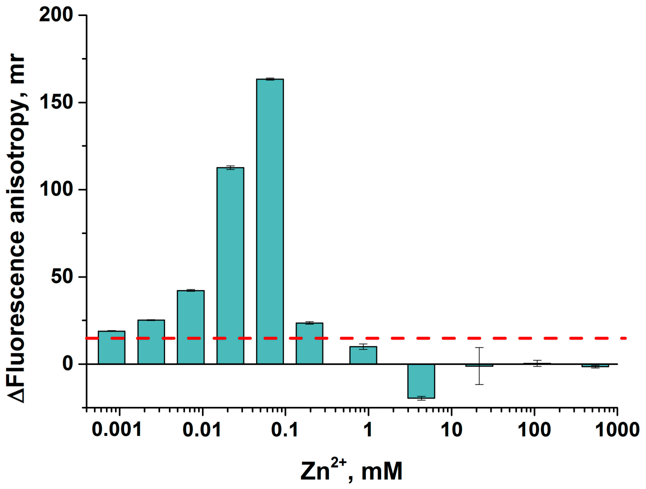

b.

Figure S5. Fluorescence anisotropy difference between STR-probe and probe at different concentrations of Zn^{2+}. (a) For L-T10 probe, (b) for L-C10 probe. The dashed line indicates ΔFA in the absence of Zn^{2+}.

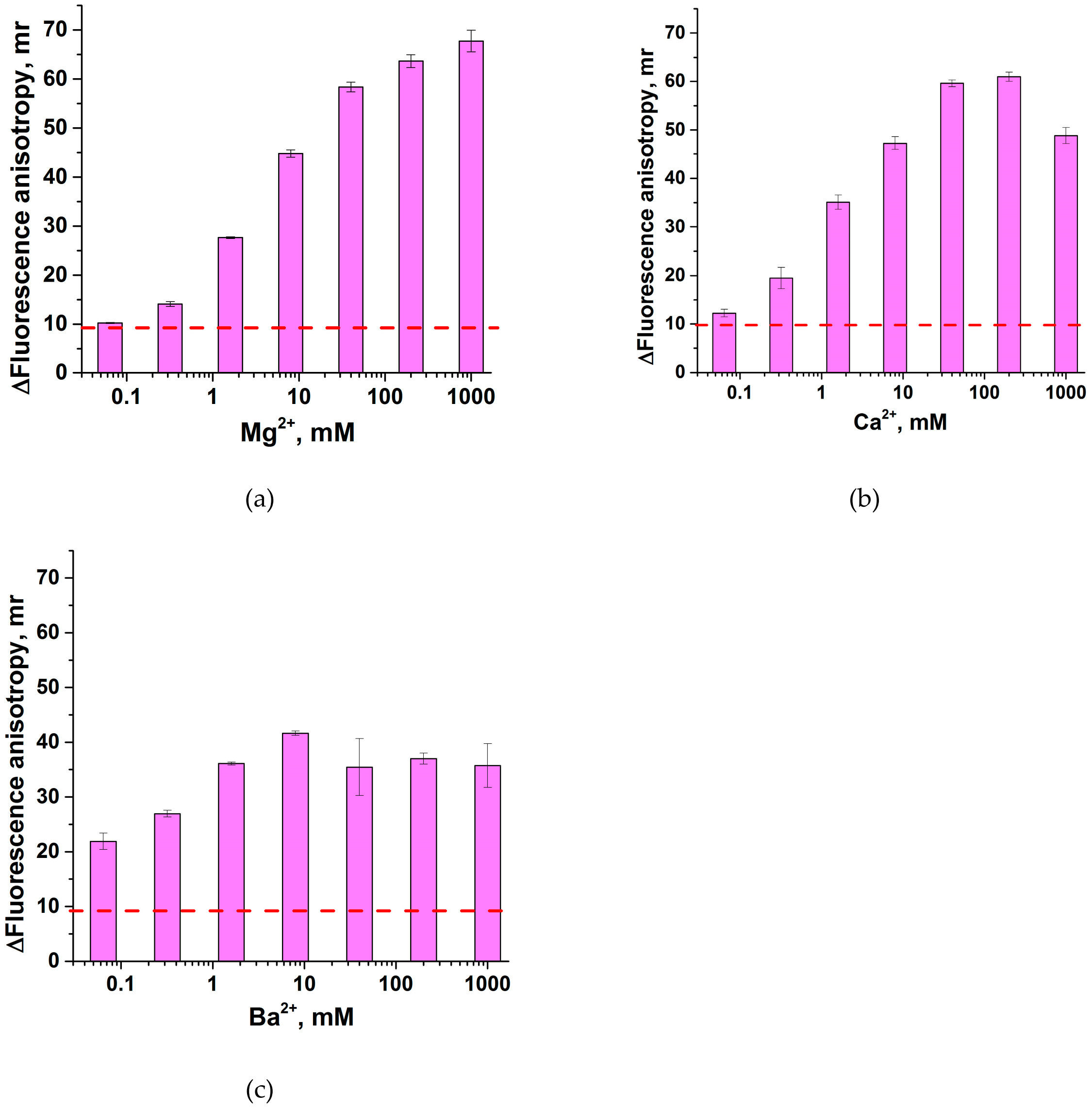

Figure S6. Impact of divalent metal ions on fluorescence anisotropy (FA) of L-A10 probe and probe with STR. The difference in FA of SRT-probe and probe on the concentration of Mg^{2+} (**a**), Ca^{2+} (**b**), and Ba^{2+} (**c**). The dashed lines indicate ΔFA in the absence of Me^{2+}.

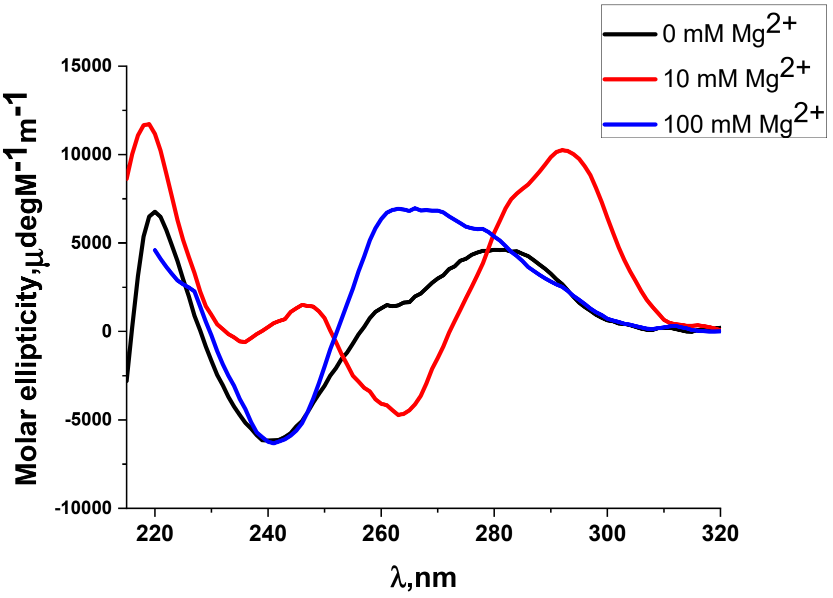

Figure S7. CD spectra of G-quadruplex carried out in 50mM Tris-HCl buffer, pH 9 at different concentrations of Mg(CH3COO)2. The reagent concentrations are 1 μM for all spectra.

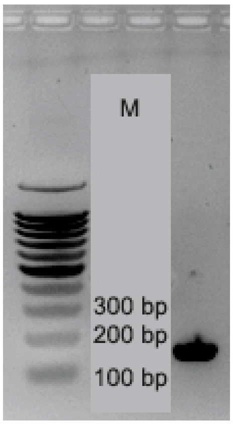

Figure S8. Scan of gel electrophoresis in 2% agarose gel of fragment of target AMY1267 gene of *Erwinia amylovora*. M – dsDNA ladder.

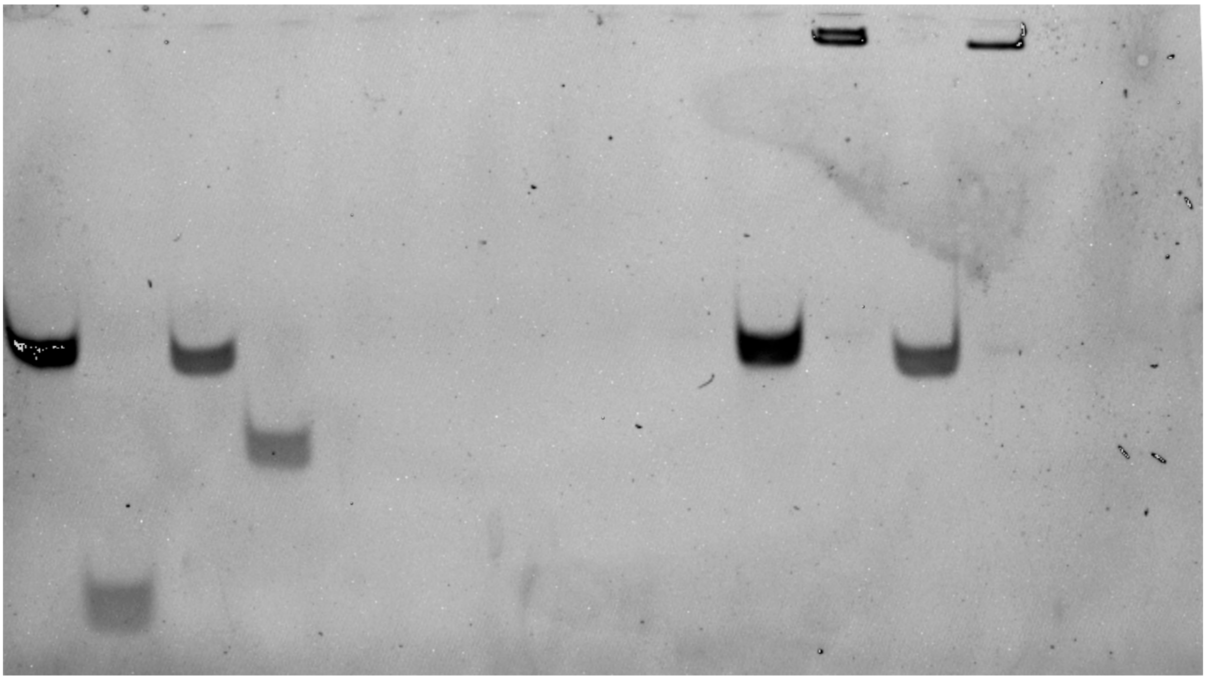

Figure S9. Scan of polyacrylamide gel after electrophoresis of ssDNA probes (1 – G-quadruplex, 2 – L -C10, 3 – H-R21, 4 – H-C10), mixtures after Cas12a-based assay with 10 nM dsDNA and ssDNA probes (5 – G-quadruplex, 6 – L -C10 + STR after assay, 7 – H-R21, 8 – H-C10 + STR before assay, 9 – without probe), mixtures after Cas12a-based assay without dsDNA and with ssDNA probes (10 – G-quadruplex, 11 – L -C10 + STR after assay, 12 – H-R21, 13 – H-C10 + STR before assay, 14 – without probe).

Table S1. Sequences of primers and guide RNA used in this research.

Name	Sequence 5′-3′	Purpose
F-PCR	CCGTGGAGACCGATCTTTTA	Forward primer for PCR
R-PCR	AAGTTTCTCCGCCCTACGAT	Reverse primer for PCR
F-RPA	GCTCTCATTGCCGTGGAGACCGATCTTTTA	Forward primer for RPA
R-RPA	TTATAACAAAAGTTTCTCCGCCCTACGAT	Reverse primer for RPA primer R
gRNA	UAAUUUCUACUAAGUGUAGAUAGAGAG GCAGCAUUCGACGAAC	gRNA for *E. amylovora* recognition
